# The Effect of Type-I Photoinitiators on the Kinetics of the UV-Induced Cotelomerization Process of Acrylate Monomers and Properties of Obtained Pressure-Sensitive Adhesives

**DOI:** 10.3390/ma14164563

**Published:** 2021-08-13

**Authors:** Agnieszka Kowalczyk, Mateusz Weisbrodt, Beata Schmidt, Agata Kraśkiewicz

**Affiliations:** Department of Chemical Organic Technology and Polymeric Materials, Faculty of Chemical Technology and Engineering, West Pomeranian University of Technology in Szczecin, 70-322 Szczecin, Poland; mateusz.weisbrodt@zut.edu.pl (M.W.); bschmidt@zut.edu.pl (B.S.); kraskiewiczagata@gmail.com (A.K.)

**Keywords:** pressure-sensitive adhesives, radical photoinitiators, telomerization, bulk photopolymerization, acrylic syrups, adhesion

## Abstract

A new method of solvent-free acrylic pressure-sensitive adhesives (PSAs) based on UV-induced cotelomerization products was presented. The key acrylic monomers (i.e., n-butyl acrylate and acrylic acid) with copolymerizable photoinitiator 4-acrylooxybenzophenone in the presence of a selected chain transfer agent (tetrabromomethane, TBM) were used in the UV-cotelomerization process. Moreover, two kinds of UV-photoinitiators (α-hydroxyalkylphenones, HPs and acylphosphine oxides, APOs) were tested. Photo-DSC, viscosity, thermogravimetric, and GPC measurements for cotelomers were performed. The kinetics study revealed that the systems with APOs, especially Omnirad 819 and Omnirad TPO, were characterized by a much higher reaction rate and greater initiation efficiency than HPs systems were. Additionally, the APO-based syrups exhibited a higher solid content (ca. 60–96 wt%), a higher dynamic viscosity (5–185 Pa·s), but slightly lower molecular weights (M_n_ and M_w_) compared to HP syrups. However, better self-adhesive features (i.e., adhesion and tack) were observed for PSAs based on cotelomers syrups obtained using APOs with lower solid contents (55–80 wt%). It was found that as the solids content (i.e., monomers conversion) increased the adhesion, the tack and glass transition temperature decreased and the type and amount of photoinitiator had no effect on polydispersity. Most of the obtained PSAs were characterized by excellent cohesion, both at 20 °C and 70 °C.

## 1. Introduction

Finding new techniques of materials joining (including the creation of thin-walled structures) is an important task for scientists and industry in order to ensure high functionality of the structure while respecting the principles of environmental protection [1]. This global trend also includes new, fast, and environmentally friendly methods of adhesives preparation, such as pressure-sensitive adhesives (PSAs). PSAs are a special class of adhesive materials used in many industrial assembly operations and most consumer applications [2,3]. PSAs do not harden but remain permanently sticky. They create a strong bond with the surface with only finger pressure and do not require activation by heat or water [4,5]. The most widely used are acrylic PSAs [6]. They are obtained predominantly by organic solvent radical polymerization or emulsion polymerization [7,8,9]. In both cases, the solvent (organic or water) must be removed in order to obtain a self-adhesive film. Increasingly, in the last decade, publications on ecological methods of PSAs preparation have been made, of which the most important are photopolymerization processes. Back et al. reported on a new pro-ecological method of obtaining solvent-free PSAs via visible-light-driven photocatalytic radical polymerization [10] and photoredox-mediated radical polymerization [11]. Instead, Beak at al. showed the free radical bulk photopolymerization process as a method of obtaining acrylic syrups to prepared PSAs [12,13,14,15,16,17]. However, the resulting reaction products (prepolymers/syrups) had a high content of unreacted monomers, so the PSA film was UV-cured in the presence of volatile compounds and must be covered with a transparent release film to avoid oxygen inhibition. Moreover, the above-mentioned publications did not show the influence of various factors on the course of the bulk photopolymerization process.

Generally, the role of the photoinitiator (PI) in radical photopolymerization processes is crucial next to the source and dose of UV-radiation, exposure time, or the influence of oxygen. However, the effect of PIs on the course of the free radical bulk photopolymerization process has not been revealed so far. Gziut at al. revealed the influence of the type and amount of PIs on the free radical bulk photopolymerization processes of butyl acrylate, glycidyl methacrylate, and 2-hydroxyethyl acrylate. The received acrylate syrups were used to obtain structural adhesive tapes [18]. In principle, photoinitiators for radical polymerization are classified as cleavage (type I) and H-abstraction-type (type II) initiators [19]. Type I photoinitiators are mostly aromatic carbonyl compounds, e.g., benzoin and derivatives, benzile ketales, acetophenones, α-hydroxyalkylphenones (HPs), and acylphospine oxides (APOs). The choice of a particular PI largely depends on the requirements of a specific application, taking into account the advantages and disadvantages of the selected PI. For example, APOs have been used in high pigmented formulations, where irradiation at longer wavelength is desired. The absorption of APOs (350-380 nm) match the emission spectrum of the main commercial light sources [20]. Furthermore, APOs are attractive to use because of their thermal stability and high reactivity of formed phosphonyl radicals. On the other hand, HPs have a strong absorption in the short-wave UV (230–270 nm) and weak absorption at longer wavelengths (up to 360 nm) and can lead to deep curing using a medium-pressure lamp. Therefore, the HPs are very versatile and efficient photoinitiators [21]. A lot of work has recently been dedicated to a new system of free radical photopolymerization initiations. New type I and type II photoinitiators based on 2,2-dimethoxy-2-phenylacetophenone, benzophenone, and thioxanthone were proposed for the free radical polymerization of acrylates [22], carbazole derivatives for LED polymerization [23], carbazole phosphine oxides [24,25], and novel-water soluble polymeric photoinitiators for acrylate polymerization [26]. However, to the best of the authors’ knowledge, there is no information in the literature about the most effective photoinitiators for the UV-initiated telomerization process.

This article is a continuation of the recent research presented on a new solvent-free method of PSA preparation for various applications [27], i.e., UV-induced cotelomerization process. Telomerization is a reaction of a polymerizable compound (M) in the presence of a chain transfer agent (YZ), which is provided to macromolecules Y (M)_n_Z, where *n* is less than 100 but is not 1 [28]. Previously, we proved that the UV-induced cotelomerization process is an environmentally friendly and fast method of obtaining pressure-sensitive adhesives (PSAs) and the effect of a functional monomer (i.e., acrylic acid) was checked. Based on these studies, the optimal composition of the monomer mixture (*n*-butyl acrylate, acrylic acid, and 4-acrylooxybenzophenone) for UV-cotelomerization was selected. Our previous and unpublished research showed that the key parameter influencing the self-adhesive properties of acrylic PSAs based on cotelomerization products (acrylic syrups, i.e., mixture of linear cotelomers and unreacted monomers) is the solid content (SC), which corresponds to the monomer’s conversion. One of the factors affecting the SC values is the type and amount of PI used for initiation of the UV-cotelomerization process. This study presented the influence of commercially available type I photoinitiators (α-hydroxyketones- HPs and acylphospine oxides—APOs) on the course of the UV-cotelomerization process, properties of reaction products (cotelomer syrups), and selected self-adhesive features of solvent-free PSAs. The new method of receiving solvent-free PSAs presented here is noteworthy as it allows one to obtain adhesive binders with a low content of unreacted monomers in a short time (30 min or even less), which was incorporated into the polyacrylate structure during the UV crosslinking stage (in the presence of crosslinking monomer and without special screens to protect against the negative effects of oxygen). The resulting PSAs can be used for the production of masking tapes, self-adhesive labels, or other self-adhesive materials.

## 2. Materials and Methods

### 2.1. Materials

The following components were used for the preparation of BA/AA/ABP cotelomers: *n*-butyl acrylate (BA), acrylic acid (AA) (BASF, Ludwigshafen, Germany) as monomers; 4-acryloylooxybenzophenone (ABP, Chemitec, Scandiccy, Italy) as copolymerizable photoinitiator; tetrabromomethane (TBM; Merck, Warsaw, Poland) as telogen; and different type I radical photoinitiators:

(1) α-Hydroxyalkylphenones (HPs): 2-hydroxy-1-(4-(4-(2-hydroxy-2-methylpropionyl)benzyl)phenyl)-2-methylpropan-1-one (Omnirad 127; IGM Resin, Waalwijk, The Netherlands) and 1-hydroxycyclohexylphenyl ketone (Omnirad 184, IGM Resins, Waalwijk, The Netherlands);
(2) Acylphosphine oxides (APOs): bis(2,4,6-trimethylbenzoyl)-phenylphosphineoxide (Omnirad 819; IGM Resins, Waalwijk, The Netherlands); 2,4,6-trimethylbenzoyl-diphenyl phosphine oxide (Omnirad TPO; IGM Resins, Waalwijk, The Netherlands) and 2,4,6-trimethylbenzoyldi-phenylphosphinate (Omnirad TPOL; IGM Resins, Waalwijk, The Netherlands);
(3) Mixture of acylphosphine oxides (APOs blend): ethyl phenyl(2,4,6- trimethylbenzoyl)phosphinate (ca. 95 wt%) and phenyl bis(2,4,6-trimethylbenzoyl)-phosphine oxide (ca. 5 wt%) (Omnirad 2100; IGM Resins, Waalwijk, The Netherlands).

The structures of photoinitiators are shown in Table 1.The components were applied without purification. A hydroxyl-terminated polybutadiene resin Hypro 1200X90 HTB (CVC Thermoset Specialties, Emerald Kalama Chemical, Kalama, WA, USA) as a multifunctional monomer of the UV-crosslinking process was used, as previously reported [27].

### 2.2. Synthesis and Characterization of BA/AA/ABP Cotelomers

**Figure 1:**
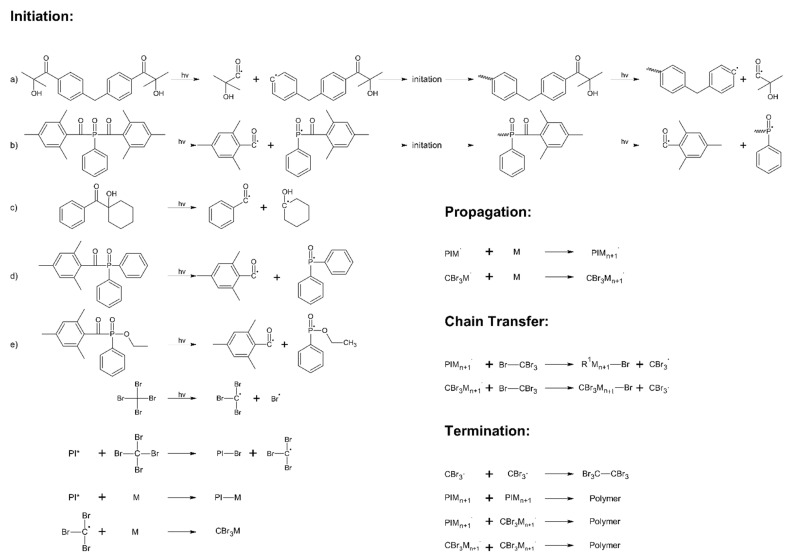
Schematic graph of UV-induced cotelomerization process with different type I radical photoinitiators.

The cotelomerization processes of BA, AA, and ABP were initiated using different radical photoinitiators (15, 30, or 45 mmol per 50 g of monomers mixture) and tetrabromomethane (2.5 phr) as telogen was used. The reaction mechanism is presented in Figure 1. Mixtures of monomers and sample symbols are shown in Table 2.

The cotelomerization processes were carried out at 20 °C for 30 min in a glass reactor (250 mL), equipped with a mechanical stirrer and thermocouple, in the presence of argon as inert gas. A mixture of monomers (50 g) was introduced into the reactor and purged with argon for 20 min. A high-intensity UV lamp (UVAHAND 250, Dr. Hönle AG UV Technology, Gräfelting, Germany) as a UV radiation source was used and was placed perpendicularly to the side wall of the reactor. The UV irradiation inside the reactor (15 mW/cm^2^) was controlled with UV-radiometer SL2W (UV-Design, Brachttal, Germany). The reactor was water-cooled (using room-temperature water).

At the beginning, the kinetics studies of the UV-induced cotelomerization process of BA, AA, and ABP (compositions in Table 2) were tested using a differential scanning calorimeter with a UV attachment (photo-DSC, DSC Q100, TA Instruments, New Castle, DE, USA; UV-light emitter Omnicure S2000; Excelitas Technologies, USA) at room temperature (isothermal measurement). Samples (5 mg) were irradiated with UV in the range 320–390 nm with an intensity of 15 mW/cm^2^ in an argon atmosphere. All DSC photopolymerization experiments were conducted in triplicate. The polymerization rate (R_p_, %/s) was calculated according to Equation (1), and the conversion of double bonds (p, %)—according to Equation (2) and photoinitiation index (I_p_)—was calculated according to Equation (3) [29].

(1) $$R_{p}=\frac{\Delta H_{t}/\mathrm{dt}}{H_{0}}$$

(2) $$p=\frac{\Delta H_{t}}{H_{0}}\;\times \;100\%$$

(3) $$I_{p\;}=\frac{R_{p}^{\max}}{t_{\max}}$$

where: dH/dt—heat flow in the polymerization reaction; H_0_—theoretical heat for the complete degree of conversion (for acrylates: ΔH = 78.0 kJ/mol); ΔH_t_—the reaction heat evolved at time t.

Then, the physicochemical properties of the BAA syrups and BAA cotelomers obtained after the bulk UV-induced cotelomerization process were examined.

Dynamic viscosity of the BAA syrups (BAA cotelomers with unreacted monomers) was measured at 25 °C by means of a DV-II Pro Extra viscometer (spindle #6, 50 rpm; Brookfield, New York, NY, USA). The solid content (SC) of the prepared syrups was determined using a Moisture Analyzer MA 50/1.X2.IC.A (Radwag, Radom, Poland). Samples (ca. 2 mg) were heated in aluminum scale pans at a temperature 105 °C for 4 h. The SC parameter was calculated according to Equation (4):

(4) $$\mathrm{SC}=\frac{m_{2}}{m_{1}}\cdot 100\;\left(\mathrm{wt}\%\right)$$

where: m_1_—initial weight of a sample; m_2_—residual weight after an evaporation process.

Gel permeation chromatography (GPC) was used to determine the molecular masses (Mw, Mn) and polydispersity (PDI) of the BAA cotelomers (post-reaction mixtures were dried at 140 °C for 4 h before the test to remove unreacted monomers); the GPC apparatus contained the refractive index detector (Merck Lachrom RI L-7490, Abingdon, UK), pump (Merck Hitachi Liquid Chromatography L-7100, Abingdon, UK) and interface (Merck Hitachi Liquid Chromatography D-7000, Abingdon, UK), and the Shodex Ohpak SB-806 MQ column with Shodex Ohpak SB-G pre-column (Merck Hitachi Liquid Chromatography L-7100, Abingdon, UK). The GPC tests were performed using polystyrene standards (Fluka and Polymer Standards Service GmbH, Mainz, Germany) in tetrahydrofurane.

### 2.3. Preparation and Characterization of Obtained Pressure-Sensitive Adhesives (PSAs)

**Figure 2:**
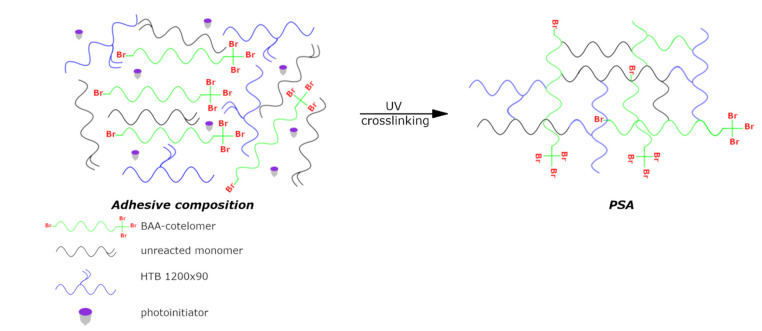
Preparation steps of the pressure-sensitive adhesives from BAA cotelomers.

The adhesive compositions for the preparation of PSAs were compounded using BAA syrups (90 wt%), HTB crosslinking agent (7.5 wt%), and TPOL photoinitiator (2.5 wt%). The PSA components were mixed using a high-speed mechanical mixer (T10 Basic Ultra-Turrax, IKA, Königswinter, Germany). The compositions were applied onto a polyester foil (50 µm) and UV-irradiated using a medium-pressure mercury lamp (UV-ABC; Hönle UV-Technology, Gräfelfing, Germany). The UV doses were 4 J/cm^2^. The UV exposition was controlled with a radiometer (Dynachem 500; Dynachem Corp., Westville, IL, USA). The basis weight of samples was 60 g/m^2^. Preparation steps of PSAs are graphically presented in Figure 2.

Self-adhesive tests (adhesion to a steel, tack, and cohesion at 20 °C and 70 °C) were performed at 23 ± 2 °C and 50 ± 5% relative humidity. Adhesion is defined as the force value required to remove pressure-sensitive material from a stainless-steel plate; the removal proceeded at an angle of 180° with a speed of 300 mm/min. Adhesion to a steel substrate was tested according to AFERA standards (Association des Fabricants Europe’ens de Rubans Auto-Adhe’sifs), i.e., AFERA 5001. Tack is characterized by a force value required to separate the stainless-steel plate and adhesive tape applied under low pressure for 0.5 s. The tack measurements were performed according to AFERA 5015. The mentioned tests were carried out with the strength machine Zwick Rolell Z010 (Zwick/Roell, Ulm, Germany). Cohesion (i.e., static shear adhesion) describes the time needed to shear off the adhesive tape sample (under load of 1 kg) from the defined steel surface. The cohesion tests were performed according to FINAT FTM 8. These parameters were evaluated using three samples of each adhesive film.

Differential scanning calorimetry (DSC Q100, TA Instr., New Castle, DE, USA) was used to determine the glass transition temperature (T_g_) values of the UV-crosslinked PSAs. Hermetic aluminum DSC pans were used and samples (ca. 10 mg) were analyzed from −80 °C to 250 °C (heating rate of 10 °C/min).

## 3. Results

### 3.1. Kinetics Study of Photo-Cotelomerization Process

**Figure 3:**
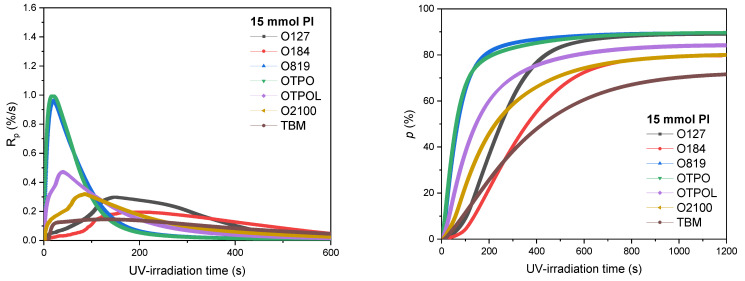

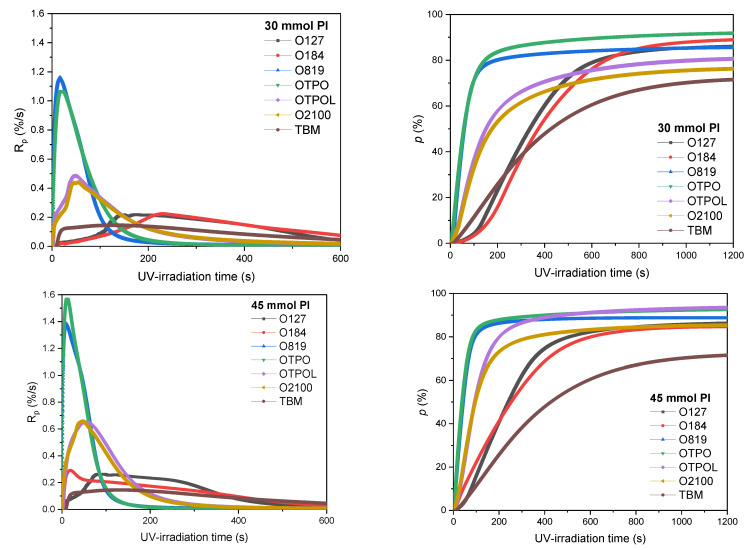
Kinetics parameters of BA, AA, and ABP photo-cotelomerization process in the presence of TBM as a chain transfer agent (I_0_ = 15 mW/cm^2^; 320–390 nm); R_p_—reaction rate; p—conversion of double bonds.

Among the described photopolymerization processes, two general types can be distinguished, namely processes taking place in thin or thick layers. Relatively few publications have dealt with bulk photopolymerization processes with mechanically mixed monomers [18,30]. To the best of the authors’ knowledge, this publication is the first to describe the effect of the type (and amount) of radical photoinitiator on the bulk photo-cotelomerization process of acrylic monomers. So far, there is a general agreement on the division into two main groups of type I photoinitiators (HPs and APOs), namely, HPs are dedicated to thin films (surface photocrosslinking), and APOs are more suitable for the photopolymerization of thick layers [21]. However, the influence of photoinitiators on UV-induced cotelomerization processes of acrylic monomers has not been investigated. A characteristic property of the process is the presence of radicals from the photolysis of telogen in the system (apart from the radicals derived from the photoinitiator photolysis and the resulting monomer radicals and macroradicals). Additionally, there are more of these radicals in the system compared to those coming from the photoinitiator. Some of the radical reactions are outlined in Figure 1. The results of the kinetics studies are presented in Figure 3 (photo-cotelomerization rate, R_p_ and monomers conversion, p).

The UV-induced cotelomerization reaction without photoinitiator (brown line) ran at a very slow rate (ca. 0.1%/s) and slowed down after about 400 s of UV-irradiation. As can be seen, as the photoinitiator content increased, the reaction rate (R_p_) values increased. Interestingly, three characteristic areas could be marked on the graphs showing the reaction rate. The first area related to the R_p_ in systems with O819 and OTPO photoinitiators, where the R_p_ values were highest and increased from ca. 1 to 1.6%/s with the PI dose. The second area was for systems with OTPOL and O2100 photoinitiators, where R_p_ values ranged from 0.3 to 0.5 %/s at the lowest PI dose and only slightly increased to about 0.6%/s at the highest PI dose. As is known, the photoinitiator O2100 contains mainly OTPOL (95 wt%); therefore, the differences between the two systems were not large, and they were revealed only at the lowest dose of PI (15 mmol). The reaction rate was also very similar at higher PI concentrations (30 or 45 mmol). The third region relates to the photo-cotelomerization rate in HP systems, for which the reaction rate was two or three times higher than in the system without any photoinitiator (i.e., 0.2 or 0.3%/s), and it increased indefinitely with the concentration of the photoinitiator. However, the reaction rates were very low compared to systems with O819 or OTPO.

**Figure 4:**
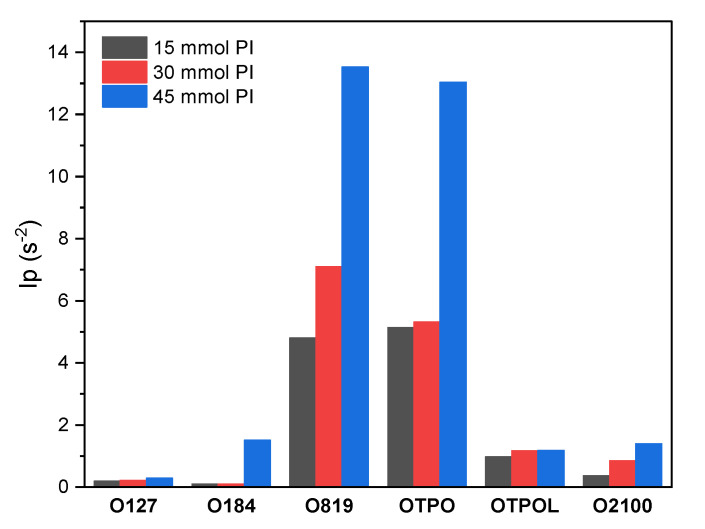
Initiation efficiency (I_p_) of the radical photoinitiators used for photo-cotelomerization processes of BA, AA, and ABP with TBM.

Moreover, some delay in the initiation of reactions in HP systems can be observed, especially at their low concentration. The maximum rate of reaction was achieved after about 150 s of exposure to UV for doses of 15 or 30 mmol of HPs, while for the system with APOs—after just a few seconds. In general, the monomer conversion exceeded 70% despite the slow reaction rates in some systems. The monomer conversion in the reference sample (without PI) was 72% after 1200 s of UV-irradiation during the photo-DSC test (without mixing of monomers mixture). High monomer conversions were obtained in the O819 and OTPO systems (90–93%), corresponding to the highest values of reaction rates, and high conversion values (over 80%) were received after 200 s of irradiation. OTPOL was second in terms of the speed of achieving the highest possible monomer conversion (80–94%), and HP was third (although, at 30 mmol PI, the final monomer conversion value was higher than for the OTPOL-based samples). The relatively high monomer conversion in the HP-based samples, despite the slow reaction rate, resulted from keeping the reaction rate relatively constant and high (0.2–0.3%/s) for a long period of exposure to light (even above 400 s). On the other hand, in samples with APOs, the reaction was rapidly inhibited (after 100 s in the case of O819 and OTPO and 200–300 s in the case of OTPOL-based samples) as a consequence of the viscosity increase in the polymerizing system. It has been confirmed by the obtained results, as well as by the calculation of initiation efficiency (I_p_), that the efficiency of APOs in the photo-cotelomerization process was higher than that of HPs. The results are presented in Figure 4.

The initiation efficiency (I_p_) of the radical PIs increased with their concentration in the system, and extremely high values of I_p_ were obtained at 45 mmol of O819 and OTPO. The order of PI efficiency was as follows: O819 > OTPO > OTPOL > O2100 > O127 > O184. Surprisingly, the mixture of OTPOL with the most effective photoinitiator O819 (i.e., O2100) was characterized by worse properties than pure OTPOL. It might be caused by the effect of too many radicals and different types of radicals in the system, which leads to their recombination or extinction of excited states in the presence of tribromomethane and bromine radicals (resulting from TBM).

### 3.2. Properties of BAA Syrups and Cotelomers

**Figure 5:**
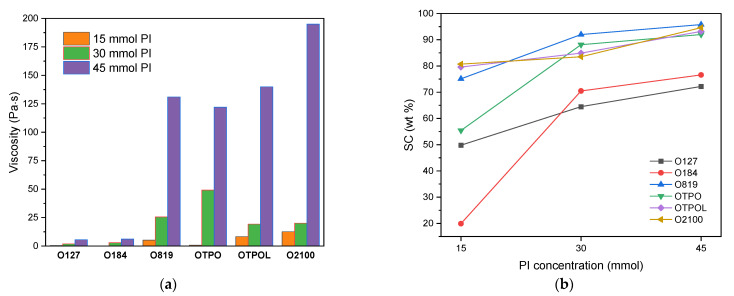
Viscosity (**a**) and solid content (**b**) of obtained BAA syrups.

The results of dynamic viscosity and solid content (SC) tests for the obtained BAA cotelomers solutions (BAA syrups) with different kinds and contents of type I radical photoinitiators are presented in Figure 5.

As can been seen, the viscosity and SC values increased with the amount of photoinitiator used in the reaction. However, HP-based syrups exhibited significantly lower viscosity values (from 0.2 to 6.1 Pa·s) than APO-based products (from 5 to 185 Pa·s). The syrups with a viscosity below 3 Pa·s (BAA-127-15, BAA-127-30, and BAA-184-15) are not suitable for coating and forming thin adhesive films (PSAs) from a practical point of view. The SC values were also relatively low for the mentioned samples, especially for BAA-127-15 and BAA-184-15 (49.9 wt% and 19.9 wt%, respectively); this confirms the lowest initiation efficiency by these photoinitiators in the tested monomers system. An additional amount of increase in HP-type photoinitiators (up to 45 mmol) only slightly increased the viscosity (ca. 6 Pa·s) and the SC values (72 and 76 wt%, respectively). In contrast with APO-type PI samples, increasing the content of PI (up to 45 mmol) caused a significant increase in viscosity of syrups (viscosity above 100 Pa·s; the samples were not suitable for coating at room temperature but—as with typical hot melt adhesives—had to be coated after heating). An extremely high viscosity value (185 Pa·s) was achieved for the sample BAA-2100-45 (with 45 mmol of Omnirad 2100). More radicals were formed in this system and the SC value reached 96 wt%. BAA syrups with APO-type PI, even with a low amount of PI (15 mmol), showed high SC values (75–81 wt%) and suitable coating viscosities (5–12 Pa·s). The BAA-TPO-15 sample (20 wt% of SC) is an exception confirming our previous research (for systems without telogen) [18]. It was noted that the highest SC values were obtained for BAA-819-45 (96 wt%). As we previously reported, the type and amount of PIs have a significant influence on the free radical bulk photopolymerization process (without telogen) of selected (meth)acrylate monomers. It has been proven that the acrylic syrups prepared using APO photoinitiators exhibited a higher viscosity and SC than samples with HPs did, as well as average molecular weights [18]. In the case of the UV-induced cotelomerization process, it turns out that samples with APOs showed lower molecular weights (M_n_ and M_w_) than samples with HPs did. The molecular weights and polydispersity of BAA-cotelomers are presented in Table 3. Higher molecular weights (M_n_ and M_w_ values) were exhibited for samples with HPs (M_n_ = 19,790–21,530 g/mol and M_w_ = 30,570–34,400 g/mol) than with APOs (M_n_ = 17,250–19,390 g/mol and M_w_ = 26,320–31,120 g/mol). Generally, the average molecular weights decreased with the amount of photoinitiator (high concentration of PI gives a high radical count and leads to the production of multiple chain polymerization reactions).

**Figure 6:**
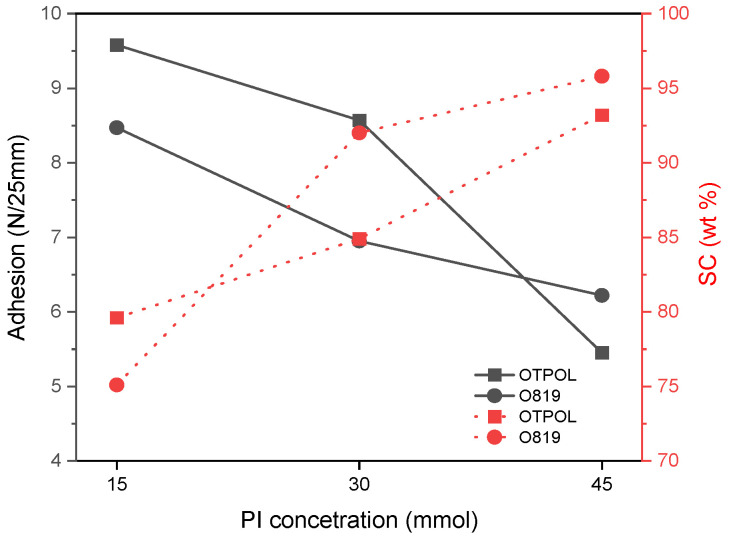
Dependence of adhesion to steel and solid content on photoinitiator concentration.

In contrast, the PDI values were very similar for all samples (1.5–1.6 a.u.). The low polydispersity index (as in polymers obtained by the ATRP method) demonstrated a near-unimodal molecular weight distribution. Additionally, it was due to the presence of the chain transfer agent (tetrabromomethane, TBM) and its interaction with photoinitiators. The several types of radicals were formed (from TBM and PIs) during UV-irradiation. The possible paths of UV-induced cotelomerization reaction are shown in Figure 1. The content of linear polymers in the system (adhesive composition contains linear BAA cotelomers, unreacted monomers, and HTB) and the average molecular weights of BAA cotelomers should influence the adhesive properties (adhesion to steel and tack) and the cohesion of the prepared PSAs. Generally, there were more linear polymers (higher SC values) with lower molecular weights in APO systems, and, conversely, fewer linear polymers but with higher molecular weights were in HP systems. The dependence of the adhesion to steel on the solids content (SC may be the same as monomer conversion [18]) is shown in Figure 6. The graph demonstrates the results only for samples BAA-819 (prepared using PI with the highest I_p_ values, Figure 4) and BAA-TPOL (PI with average I_p_ values). The adhesion values were higher for PSAs based on BAA-TPOL than BAA-819 and they decreased with the PI concentration (from 9.5 to 5.5 N/25 mm). The SC values were slightly lower for BAA-TPOL samples (80–94 wt%) than for the PI samples with the highest initiation efficiency (75–96 wt%). The SC values increased (higher content of linear polymers) as the adhesion values decreased (for both considered systems). Both systems (BAA-819 and BAA-TPOL) were characterized by very similar values of M_n_ (ca. 17,000 g/mol) and M_w_ (27,000 g/mol; Table 3).

**Figure 7:**
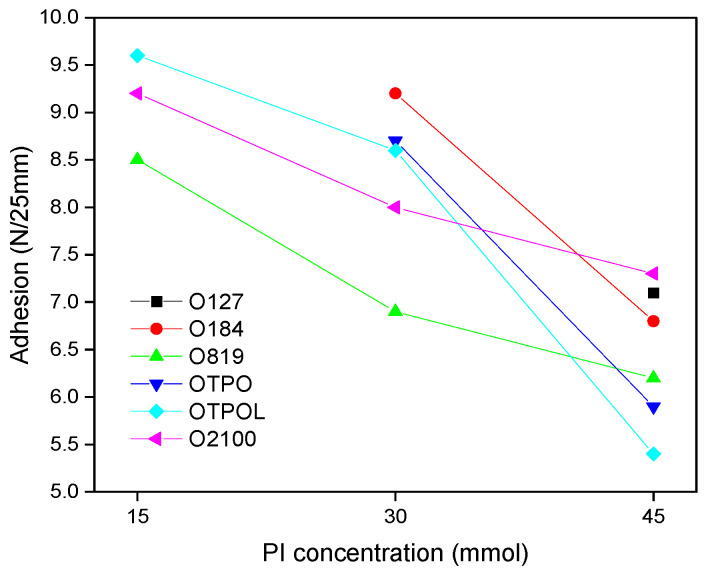
Adhesion to steel of PSA based on BAA cotelomers prepared using different types and concentrations of PIs.

However, systems with a higher SC value (higher content of linear cotelomers with very similar molecular weights) include more Br-terminated chains (according to the telomerization mechanism, the ends of the formed telomer contain fragments of telogen [28]). This likely results in less adhesion to the substrate (nonpolar cotelomer chain ends). The influence of various types and concentrations of PIs on the adhesion is shown in Figure 7).

Regardless of the PI type, with the increase in PI concentration (increase in the SC value), the adhesion of prepared PSAs decreased. The highest values of adhesion to steel were obtained for PSAs based on BAA cotelomers with the lowest dose of photoinitiator (15 mmol). The best result of adhesion was received for the PSA from BAA-TPOL-15 syrup (9.7 N/25 mm). A high result of adhesion (9.4 N/25 mm) was also noted for PSA with photoinitiator O2100. Interestingly, high adhesion values (9.2 N/25mm; PI concentration 30 mmol) were obtained for PSA prepared with the least effective photoinitiator O184 (HPs-type). However, the SC value of the BAA-184-30 syrup was 70%. It can be concluded that the SC value in the obtained syrups had a key influence on the adhesion to steel. The tack values also decreased with the concentration of photoinitiator. The results of tack measurements are shown in Table 4. The tack values were generally lower than the adhesion values (typical for PSA).

**Figure 8:**
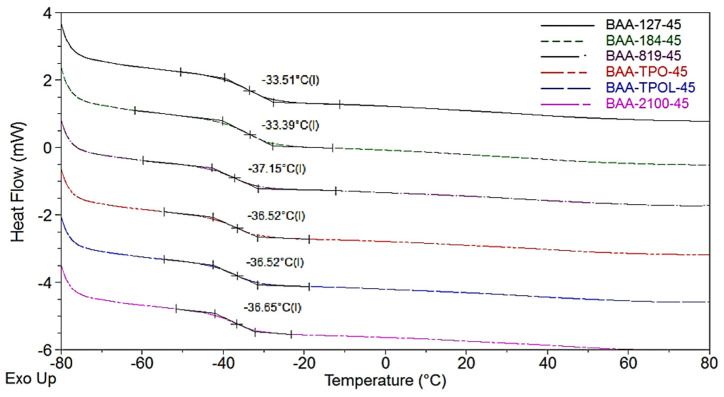
T_g_ values of PSAs based on BAA cotelomer.

The highest tack values were recorded for samples based on BAA-TPO-30 and BAA-819-15 (7.1 N and 6.5 N, respectively). Interestingly, almost all prepared PSAs were characterized by excellent cohesion at 20 °C (100 h) and 70 °C (72 h). Only samples with low tack values (usually ca. 5 N or less, with APO-type PI concentration—45 mmol) did not wet the steel surface sufficiently during the cohesion test. Additionally, these samples obtained a low cohesion result. Interestingly, samples from HP-type PIs (O127 and O184), even at a low tack value (2 or 3 N), were characterized by very high cohesion due to the presence of cotelomers with higher molecular weights compared to the PSA with APOs (Table 2). Molecular weight values and T_g_ values are critical for the use of a polymer as a PSA. The T_g_ values for selected PSAs (with 45 mmol of PI) are shown in Figure 8.

As can be seen, PSAs possessed the T_g_ range from ca. −33 °C (PSAs with HPs) to ca. −37 °C (PSAs with APOs). It is known that PSAs should have a T_g_ of about −15 to −5 °C or less, and acrylic PSA possesses a T_g_ range from −40 °C to −60 °C. The T_g_ is a function of molecular weight (M_n_), as well as other chemical and macromolecular characteristic, e.g., chemical composition and crosslink density [31]. It should be noted that the BAA cotelomers with HPs (45 mmol) exhibited higher M_n_ values (ca. 20,000 g/mol; Table 3) than the BAA with APOs (ca. 17,000 g/mol). Thus, the PSAs based on the BAA-HPs exhibited higher T_g_ values (less chains mobility) than samples with APOs. Additionally, the research shows that PSA with HPs was characterized by a higher crosslinking density (lower SC value, ca. 70 wt%) than samples with APOs (ca. 90 wt%); therefore, PSA-HPs possessed a higher T_g_. It is known that crosslinking reduces chain mobility and, thus, increases the T_g_. However, it can be concluded that the differences in T_g_ values were not significantly large. This is because the obtained PSAs, although they differed in crosslink density, chemically contained the same amount of polar monomer (AA) and were crosslinked in the same way (using the same amount of HTB and the same UV dose). These factors would have a greater influence on the difference in T_g_ values.

## 4. Conclusions

In this paper, a new preparation method (i.e., UV-induced cotelomerization process) of solvent-free acrylic pressure-sensitive adhesives was presented and the influence of type I radical photoinitiators (HPs and APOs) on the kinetic parameters of the process was investigated. Moreover, the physico-chemical properties of cotelomer syrups and self-adhesive features of prepared PSAs were tested. It was revealed that APOs were significantly more efficient in the cotelomerization process than HPs were. The order of PI efficiency has been proven: O819 > OTPO > OTPOL > O2100 > O127 > O184. A high monomer conversion (the solid content) was obtained even at the lowest PI concentration (15 mmol) in systems with O819 (81 wt%) and OTPO (75 wt%). It was also found that cotelomer syrups based on APOs were characterized by considerable higher viscosity values than HP-based syrups. Moreover, the APO-based syrups contained more linear polymers (high SC values) with lower molecular weights than systems with HPs. These have been found to be key factors in the self-adhesive properties of PSAs. Higher adhesion and tack values were observed at low SC (55–80 wt%) and low-molecular-weight cotelomers (i.e., with APOs). A significant reduction in adhesion and tack was observed with increasing SC values (>80 wt%). It can be concluded that the vast majority of PSAs showed excellent cohesion (>72 h), regardless of the SC value or the molecular weights of the cotelomers. However, the high cohesion is most likely due to the method of crosslinking itself and the selection of the monomer and the UV dose. The methods of adhesive composition modification (with hydroxyl-terminated polybutadiene) itself and by UV-crosslinking were correct and noteworthy. The SC values and molecular weight had a great impact on T_g_ values of prepared PSAs as well. The cotelomers with higher SC values and lower M_n_ gave PSAs with lower T_g_ values.

## Figures and Tables

**Table 1:** Structures of tested photoinitiators.

PI Type	PI Names and Chemical Structure			
HPs	Omnirad 127		Omnirad 184	
	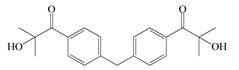		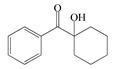	
APOs	Omnirad 819	Omnirad TPO		Omnirad TPOL
	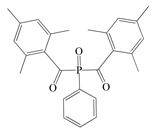	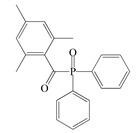		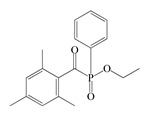
APOs Blend	Omnirad 2100 (O.TPO-L + O.819) (95/5)			
	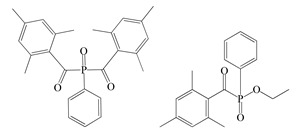			

**Table 2:** Compositions of monomers, telogen, and photoinitiators for UV-induced cotelomerization process.

Cotelomer Acronym	Monomers (wt%)			PI	
	BA	AA	APB	Symbol	mmol *
BAA-127-15	91.5	7.5	1	O127	15
BAA-127-30					30
BAA-127-45					45
BAA-184-15				O184	15
BAA-184-30					30
BAA-184-45					45
BAA-819-15				O819	15
BAA-819-30					30
BAA-819-45					45
BAA-TPO-15				OTPO	15
BAA-TPO-30					30
BAA-TPO-45					45
BAA-TPOL-15				OTPOL	15
BAA-TPOL-30					30
BAA-TPOL-45					45
BAA-2100-15				O2100	15
BAA-2100-30					30
BAA-2100-45					45

* per 50 g of monomer mixture.

**Table 3:** Molecular weights and polydispersity of BAA-cotelomers.

Cotelomer Symbol	M_n_ (g/mol)	M_w_ (g/mol)	PDI
BAA-127-15	21,530	33,770	1.57
BAA-127-30	20,520	32,930	1.61
BAA-127-45	20,100	30,570	1.52
BAA-184-15	21,010	34,400	1.64
BAA-184-30	21,060	32,540	1.54
BAA-184-45	19,790	30,670	1.55
BAA-819-15	18,150	28,440	1.57
BAA-819-30	17,650	27,090	1.53
BAA-819-45	17,460	26,930	1.54
BAA-TPO-15	19,390	31,120	1.60
BAA-TPO-30	18,210	27,190	1.49
BAA-TPO-45	17,460	26,430	1.51
BAA-TPOL-15	17,500	28,670	1.64
BAA-TPOL-30	18,260	27,450	1.50
BAA-TPOL-45	17,250	26,320	1.53
BAA-2100-15	19,100	28,990	1.52
BAA-2100-30	18,940	28,620	1.51
BAA-2100-45	17,970	27,450	1.53

**Table 4:** Cohesion and tack values of PSAs based on BAA cotelomers.

PSA Symbol	Cohesion [h]		Tack (N)
	20 °C	70 °C	
BAA-127-15	nd	nd	nd
BAA-127-30	nd	nd	nd
BAA-127-45	100 ± 3	72 ± 2	2.1 ± 0.2
BAA-184-15	nd	nd	nd
BAA-184-30	100 ± 4	72 ± 1	3.0± 0.3
BAA-184-45	100 ± 5	72 ± 2	2.5 ± 0.1
BAA-819-15	100 ± 3	72 ± 2	6.5 ± 0.3
BAA-819-30	14 ± 0.5	3 ± 0.2	5.2 ± 0.3
BAA-819-45	5 ± 0.5	0.1 ± 0.02	2.4 ± 0.2
BAA-TPO-15	nd	nd	nd
BAA-TPO-30	100 ± 5	72 ± 3	7.1 ± 0.4
BAA-TPO-45	11 ± 0.3	2 ± 0.2	5.3 ± 0.2
BAA-TPOL-15	100 ± 6	72 ± 3	3.2 ± 0.1
BAA-TPOL-30	100 ± 4	72 ± 4	3.1 ± 0.2
BAA-TPOL-45	1 ± 0.2	0.3 ± 0.2	2.5 ± 0.2
BAA-2100-15	100 ± 2	72 ± 3	5.1 ± 0.4
BAA-2100-30	100 ± 5	72 ± 3	5.0 ± 0.3
BAA-2100-45	2 ± 0.2	0.6 ± 0.2	2.5 ± 0.1

nd—no data.

## Data Availability

The data presented in this study are available on request from the corresponding author.
